# Clergymen and Physicians as Dentists

**Published:** 1849-01

**Authors:** J. W. Power


### CLERGYMEN AND PHYSICIANS AS DENTISTS.

*Messrs. Editors:*

We desire to say a few words on the propriety of members of other professions assuming the practice of dentistry, in addition to their own legitimate calling. It is scarcely necessary for us to state that the dental profession is now esteemed, by its most intelligent members, as a distinct avocation, embracing in its principles a regular science, ample enough in its scope to engage the learning and talents, and wide enough in its practical duties to employ all the time of the most skilled and indefatigable practitioner. It is also viewed in this light by many of the medical profession. You can scarcely find an intelligent physician who will not concur in the opinion, that dentistry should be a distinct calling—that it requires, in part, a different education from what is necessary in the practice of medicine, and demands the whole attention of the individual who assumes the practice of its responsible duties.

The effort which is now making, by a portion of the profession, to establish dental surgery on an independent basis, is not only approved generally by the medical profession, but also co-operated in by some of its most distinguished members. Look to the Faculties of the two dental institutions recently established in our country, and you will find them in part composed of physicians. But, without referring to the judgment of the intelligent dentist or physician, who, it is but just to suppose, is the best qualified to decide the claims which dental surgery possesses upon the time and talents of its practitioner; we find the same opinion to obtain in the community at large, among all classes who have paid any attention whatever to the subject. This is evinced by the fact that almost every individual needing the services of a dentist, will always prefer employing a regular operator, unless influenced by extraneous circumstances disconnected with the business.

Now if dentistry is thus viewed by all classes of society, professional as well as unprofessional, as a distinct calling, possessing just claims to the dignity of a science, demanding all the time and talents of an individual to master it thoroughly and practice it efficiently, is not the attempt which we see manifested by a few persons, belonging particularly to the clerical and medical professions, to append it to their own appropriate calling, obviously improper? Will not their duties inevitably clash?

Now it is not our province to descant upon the peculiar duties and responsibilities belonging to a minister of the gospel, to fix the exact boundaries of his sphere of action, to determine the quantum of learning, the degree of intellectual vigor he should possess, or the amount of time he should devote to his sacred calling. It will suffice for our purpose merely to inquire, who are the divines, in every Christian denomination, most eminent for their piety and usefulness? Are they men who have divided their attention between their own and some secular employments? On the other hand, are they not rather characterised for their exclusive devotion to their high mission—for their intense study of the *Scriptures*—for the deepest draughts at its fountains of wisdom and learning—for their ever active and indefatigable labors in disseminating the *gospel* they profess to teach? In promoting the cause of that *Master* in whose service they have engaged, have they not found an object worthy of their highest ambition—a field of action ample enough in its scope to engage the loftiest powers of their intellect, and intimate enough in its relations to the interests of humanity to elicit the warmest, deepest, holiest sympathies of the soul? And after a life wholly spent in that *service*, adorned by the most eminent piety and crowned with the brightest trophies of success, do they not rather feel as if the work *He* commissioned them to do is but imperfectly performed? Do they not rather mourn over many a feeble effort, many a duty undischarged, many an opportunity of doing good neglected?

If such then be the character, the practice, and feelings of the most distinguished members of the clerical profession, a reference to its intrinsic importance is wholly unnecessary to determine that the faithful performance of its duties is incompatible with the practice of dental surgery. What apology then can be offered by those who are endeavoring to combine them together. Perhaps their views in relation to the triumphs of the *gospel* are more enlarged than the limited visions which come within the comprehension of ordinary minds—in their estimation the *kingdom* of *darkness* may be nearly vanquished, the millenium about to be ushered in—there may, therefore, be no longer any necessity for active warfare—the soldier of the cross may now ground his arms, and relax at least a portion of his vigilance. He may now turn from the anxieties, the toils and dangers of the conflict, and indulge in the more pleasing idea of relaxation and worldly ease. He may sip a little at the cup of pleasure, and appropriate to his own benefit a few of the loaves and fishes belonging to other professions; or perhaps their intellect is of such a gigantic order, that it is capable of grasping in its comprehensive range a wider sphere of action than belongs to the routine of a single profession. Difficulties, which may appear insurmountable to common men, to them may seem as "trifles light as air."

Now we would ask, in all sincerity, these individuals who are attempting to tarnish the ermine of this high and holy office by collision with secular employments, is it not a mercenary spirit which induces you to thus amalgamate two vocations wholly incompatible with each other? Is it not a desire to obtain a little more of this world's gear—to enjoy a few more of the luxuries of life—to appear in a newer garb, with a better equipage—to have your pockets lined with a little more of the *needful?* You have perhaps found the business of preaching, as far as the emoluments are concerned, rather a barren field; it probably too literally fulfils the homely adage, "All talk and no cider," for your notions of ease and gentility; I will, therefore, look around me, and see if I cannot discover some method by which I can relieve this manifest sterility. Dentistry, I find, will admirably answer the purpose. It is rather an easy, profitable business, requiring, in my estimation, no great skill or science to practice it—at all events, I can operate among the *brethren*, for a little less, perhaps, than the ordinary charge. I can thus be sure of obtaining their custom, while their charity for my character as a minister of the gospel will certainly cover over any imperfection in my operations. It will not materially encroach upon my ministerial duties. I frequently possess a leisure hour. I perhaps spend too much time in pastoral visitations, and in the preparation of my sermons. These I can curtail without any disparagement to my usefulness—at any rate, the additional funds I can raise in this way are absolutely necessary, as I desire to purchase a new piece of property; my family is growing, and my children must be educated.

Have you endeavored to find, in some such reflections as these, an apology for your course? But have not the irrepressible whisperings of conscience told you that it is futile? Or has no sage, reflecting member of your church ventured to suggest, in a mild admonitory manner, that the vocation of a dental surgeon might possibly be inconsistent with the higher obligations you have assumed? As you have advanced in the exercise of the combined duties of these two professions, you have probably discovered that you were entirely mistaken as to the nature of dentistry. You have found that its skilful practice requires a far more extensive preparatory training than you obtained—a greater knowledge of *collateral science* than you possess, or can well acquire. You have found its business sadly encroaching on that portion of your time allotted to your ministerial duties, breaking in upon the hallowed hours that should be devoted to the services of the sanctuary, to the social prayer meeting, to the chamber and the couch of the sick and the dying of your flock, absorbing more and more of your attention, gradually effacing from your bosom the vivid impression of spiritual things, and threatening to completely secularize your mind. Perhaps, on the eve of preparation for the pulpit, a patient appears, requesting a dental operation. The job is rather an important one, and must be performed immediately. You cannot well let it slip your hands. With some compunction of conscience you undertake it, and leave the performance of a far more important duty to depend upon the mere impulse of the moment. How cold, how powerless, fall the words from your lips! Your congregation go from the church wearied and listless, unrefreshed by the fervid outpourings of a soul that has imbibed its eloquence at the fountain of heavenly inspiration. The church is not built up. The cause of *Zion* languishes in your hands, your labors become barren and lifeless, and soon perhaps you are compelled to seek some other field for your operations.

Now, if you are influenced by a benevolent desire to benefit your fellow-men, as we trust you are, let us entreat you to abandon this course, alike inconsistent with the character of a Christian minister, and the reputation of a scientific and high-minded dental operator. If you sincerely believe it to be your duty to preach the gospel, and possess the education and talents, and that other qualification, most important of all, true piety, which are necessary to make you useful in that high vocation, let us say to you, persevere undeviatingly in its path, and perform with your might what your hand findeth to do, and you have the promise of an *infinite Being*, whose power and beneficence can never fail, that all these things, the comfortable appliances of life, will be added unto you. But, on the other hand, if you have found that you have mistaken your proper calling—that you lack the piety and talents of an acceptable preacher—that the church is about to spew you from its bosom as a profitless, time-serving laborer, come out from its midst, and let the clerical mantle you have lifted with unhallowed hands fall on a more worthy head. Stand up before society in your native garb, and, with the spirit of a man, endeavor to obtain an honest livelihood by the practice of dentistry alone, or some other honest calling.

The other branch of our subject, the propriety of physicians assuming the practice of dentistry in addition to their own profession, we must defer to a future article.

J. W. POWER.

